# Delivering proportionate governance in the era of eHealth: Making linkage and privacy work together

**DOI:** 10.1177/0968533213508974

**Published:** 2013-06

**Authors:** Nayha Sethi, Graeme T. Laurie

**Affiliations:** University of Edinburgh, UK

**Keywords:** Information governance, proportionality, principles, data linkage, eHealth, research

## Abstract

This article advances a principled proportionate governance model (PPGM) that overcomes key impediments to using health records for research. Despite increasing initiatives for maximising benefits of data linkage, significant challenges remain, including a culture of caution around data sharing and linkage, failure to make use of flexibilities within the law and failure to incorporate intelligent iterative design. The article identifies key issues for consideration and posits a flexible and accessible governance model that provides a robust and efficient means of paying due regard to both privacy and the public interests in research. We argue that proportionate governance based on clear guiding principles accurately gauges risks associated with data uses and assigns safeguards accordingly. This requires a clear articulation of roles and responsibilities at all levels of decision-making and effective training for researchers and data custodians. Accordingly, the PPGM encourages and supports defensible judgements about data linkage in the public interest.

## Introduction

The linking of health records and other data for research has great potential to bring about considerable improvements in the health and well-being of populations.^1^ Initiatives dedicated to maximising the benefits of data linkage, both within and beyond the health sector, have already made significant progress.^2^ Models in Western Australia^3^ and Manitoba^4^ have proven particularly successful. The United Kingdom is also developing initiatives, with strong encouragement from the government and significant financial support of the major funding councils (Research Councils UK). The most recent £19m collaborative funding call was an unprecedented endeavour involving all established funding councils, Wellcome Trust, health charities and regional government offices. It stressed that ‘ … it is vital that the UK research community is in a strong position to maximise the health research potential offered by linking electronic health records with other forms of routinely collected data and research datasets’.^5^ The era of eHealth is truly upon us.^6^

The data linkage initiatives already existing in the United Kingdom^7^ have reinforced very clearly the inadequacies of the current governance framework and its inability to meet the needs of pre-existing and future (increasingly ambitious) linkages. The regulatory hurdles obstructing the optimal use of data for research are only too familiar and have been extensively discussed within the literature.^8^ The same key challenges continually re-emerge. These are that the current landscape is primarily characterised by (i) a culture of caution among data custodians, many of whom are unwilling to link or share data, (ii) the failure to take account of flexibilities within the law that permit and support such linking and sharing and (iii) the failure of the regulatory framework to reflect or incorporate iterative intelligent design of instances of ‘good governance’.^9^

The establishment of the Health Research Authority in December 2011 was a clear signal from the UK government to deliver more streamlined governance mechanisms.^10^ This concrete action is a manifestation of the political rhetorical commitment to improvement and efficiency that followed the publication of the Academy of Medical Sciences report in 2011 and which purported to lay down the ‘guiding principles’ for advancement in this field:

to safeguard the well-being of research participants; to facilitate high-quality health research to the public benefit; to be proportionate, efficient and coordinated and maintain and, to build confidence in the conduct and value of health research through independence, transparency, accountability and consistency.^11^

But the devil is in the detail of what this can, and should, mean in practice. These ‘principles’ have the quality of self-evident truths: no one would support a system that was unsafe, obstructive, disproportionate and untrustworthy. Rhetoric aside, then, we must ask what these claims and this opportunity will mean for the future of information governance in health research.

We argue in this article that these common objectives can be achieved via the delivery of a *principled* proportionate governance model. This has the potential not only to overcome existing challenges but also to provide additional benefits to the governance framework, for example, by stressing and facilitating a holistic approach to risk assessment, which extends beyond a tick-box mentality towards responsible data sharing. In the first part of the article, we examine forensically the key obstacles within the current governance regime to reveal their true nature as part of a currently disconnected regulatory architecture. We acknowledge that whilst efforts have been made to ameliorate the situation, substantial steps must still be taken before optimal governance is achieved. Next, we offer key considerations that we argue must be taken into account when considering how to deliver proportionate governance; this includes the methods to be employed to uncover what the considerations are, notably through engagement with stakeholders. Finally, we propose a principled proportionate governance model, highlighting each of its key components and the significance of their inclusion. We use a case in point to illustrate the added value which the model brings to practical implementation of the notion of proportionate governance that is the central aspiration of the Health Research Agency (HRA) and Academy of Medical Sciences (AMS). This is the example of the Scottish Health Informatics Programme,^12^ sponsored by the Wellcome Trust,^13^ and which began operationalisation of a nationwide proportionate governance model in Scotland in 2013.

## Key obstacles within a suboptimal landscape

To state that linkage of datasets has great research potential is neither novel nor is it a phenomenon that is exclusive to the health sector.^14^ Moreover, the complex regulatory landscape around which researchers, legally responsible data controllers and others with custodian duties for handling data must navigate to facilitate and/or conduct research has also received extensive coverage.^15^ And yet, there is surprisingly little concrete discussion in the literature about how to improve the situation. The growing number of initiatives dedicated to maximising the benefits of data linkage – including, but not restricted to health, social care, environmental and education sectors – makes it imperative that the key challenges impeding research are tackled, and most particularly that traditional approaches to information governance are reconsidered.^16^

Extensive review of the current governance landscape and its challenges can be found elsewhere.^17^ The following section suggests, rather, that the key obstacles currently impeding data linkage research fall into three categories: (i) a culture of caution,^18^ (ii) a failure to take account of flexibilities within the law and (iii) a failure to build and incorporate iterative intelligent design into the regulatory framework. This serves to create a tabula rasa for considering thereafter what good governance in this field might look like. It necessitates that the challenges are correctly identified and posits that governance must be co-produced as part of a collective exercise between all stakeholders.

### Culture of caution

The complex legislative landscape governing data use for research has been subjected to sustained critique. The most common attacks centre on its overburdensome and confusing nature.^19^ Key legislation, including both the European Data Protection Directive^20^ and its UK embodiment, the Data Protection Act (DPA) 1998 are unhelpfully vague and open to varying interpretation,^21^ even in terms of basic data protection concepts.^22^ The numerous relevant legislative provisions that must be observed, not to mention the procedural requirements imposed upon data controllers and researchers, can prove both unclear and yet onerous, causing unnecessary time delays, duplication of efforts and uncertainty. Reporting on the research framework within the UK, for example, the Department of Health identified 43 pieces of relevant legislation, 12 sets of relevant standards and 8 professional codes of conduct, concluding that what ‘this has bred is a culture of caution, confusion, uncertainty and inconsistency’.^23^

The mantra of ‘culture of caution’ has proliferated within the research community (including data custodians and researchers) and has proven particularly difficult to displace. Rather than risk sanctions for misunderstanding legislative requirements, some data custodians have tended towards more conservative approaches to managing data access requests,^24^ rendering access to potentially research-rich data problematic. This has led researchers and data custodians alike to exercise ‘ … a degree of caution that may go beyond what is required within the law itself. This can apply to individual judgments around access to data, where possible solutions are not fully explored because of the perception of the barriers’.^25^ The complex landscape and ‘the many actors and interests at play, makes confidently operating (and data sharing) within the research environment very difficult’.^26^ Ironically, however, as has been pointed out by the Information Commissioner’s Office (ICO):

‘Organisations that don’t understand what can and cannot be done legally are as likely to disadvantage their clients through excessive caution as they are by carelessness.’ ^27^ Put otherwise, doing nothing is not a sustainable option when it comes to lawful and effective data management. Fear and ignorance are not excusable, and the path towards a robust data custodianship system must begin with a sound grasp of the law, and importantly, the opportunities that it affords.

### Failure to make use of flexibilities

A fact often overlooked because of the complex regulatory landscape is that flexibilities to address some of the regulatory hurdles already exist within the current framework. For example, a research exemption exists in data protection law whereby data obtained for one purpose can later be used for a research purpose so long as two crucial criteria are met:

(i) … the data must not be processed to support measures or decisions with respect to particular individuals, and (ii) the data must not be processed in such a way that substantial damage or substantial distress is, or is likely to be, caused to any data subject.^28^

If applicable, the consequence is that data can be retained indefinitely (normally data must be destroyed after original purposes for processing are met), and data subjects need not be granted access to their data (otherwise a norm in the regulations), so long as research results are not published in an identifiable form. Notwithstanding, a Code of Practice from the ICO suggests that granting such access is good practice.^29^ Moreover, the reduction in burden might be slight because, whilst consent to research uses is not mandated, data subjects must still have adequate notice of the fact that data are being used for research. And if consent is not obtained and research data cannot be published in an effectively anonymised form, then subject access must be granted lest the researcher be exposed to an action for breach of data protection (unless it could be shown that there was no alternative but to publish the results in an identifiable form).

The restrictions on this exemption are largely driven by concerns about the autonomy of the data subject. These are compounded by a related phenomenon that we have called the ‘fetishisation of consent’.^30^ This has emerged as a key issue in recent times across many areas of health law and regulation. It can be summed up in the current context as follows: there is a tendency to view consent as a *necessary* requirement for using data for research and to regard it as a panacea that alone sufficiently addresses the concerns around data use for research. This obvious fallacy aside, and acknowledging that consent may often be desirable, it is crucial to note that, in law, consent is not a requirement to render uses legal.^31^ Far less is it necessarily the optimal of available mechanisms for achieving the twin aims of protecting individual autonomy and promoting the public interest in research,^32^ nor indeed for regulating data sharing more generally.^33^ Nonetheless, in the health data context, this reliance on consent translates to a tendency to shape information governance mechanisms around the ‘consent moment’.^34^ Where this is not possible, anonymisation of data has emerged as the default. This has been referred to as the ‘consent or anonymise’ approach.^35^

Anonymisation techniques render identification or re-identification less likely but they do not, contrary to some belief, guarantee anonymity. Fortunately, the law does not require this. The UK ICO has recently released its Code of Practice on anonymisation, which emphasises that whilst anonymisation is desirable, it is not always necessary. Rather, what is paramount in these cases is the security of the data.^36^ Thus, neither the consent nor the anonymise approach is mandated nor, as we will argue, is either necessarily conducive to effective data sharing for research.

A third governance pathway is authorisation. Authorisation involves an individual or collective body making a decision about whether data should be shared (and in what form) when it is neither possible nor practicable for the data subject to input to the process and often when the data are identifiable. Caldicott Guardians,^37^ for example, are responsible for deciding whether patients’ confidential data should be used, whether or not the data need to undergo anonymisation and whether consent should be sought.^38^ Equally, the Confidentiality Advisory Group (CAG)^39,40^ in England is charged with considering research-based data access applications submitted to the HRA.^41^ It has authority under section 251 of the National Health Service (NHS) Act 2006 to advise the Secretary of State, when a study can go ahead in the public interest and using data without gaining data subject consent. Similarly, the Privacy Advisory Committee (PAC) in Scotland^42^ advises the NHS National Services Scotland (NSS) and National Registers Scotland (NRS) upon data linkages, effectively providing a form of authority to link or share data by consultation and independent oversight.

It is our opinion that authorisation has not received the attention and consideration that it merits as an alternative and complementary mechanism to consent and anonymisation. As Law notes, ‘[m]any writers are passionate about the primacy of informed consent’,^43^ with some considering that ‘the requirements to seek an individual’s consent to participate and to provide data for a specific purpose must take precedence.’^44^ We discuss the problems that arise when only relying upon consent further below, and despite the initial objections that authorisation might raise, we would argue that ultimately it represents an important governance mechanism whereby an individual or body is entrusted as a proxy decision-maker. We appreciate the cultural challenges that might be encountered in securing buy-in for authorisation-based mechanisms within the research and data sharing context. We are not, however, suggesting that authorisation should replace consent or anonymisation, but rather that it should be considered in tandem with these governance tools that can operate alone or in combination to deliver good governance across a range of possible circumstances. Moreover, in governance terms, we contend that it delivers more robust means to protect the core interests at stake than either consent or anonymisation alone.

None of these existing mechanisms can change the legal reality that ultimate responsibility for data processing rests with data controllers.^45^ This status is one that exists as a matter of fact and law, that is, if someone is acting *in fact* in the capacity as a data controller in a literal sense that they control the data and their uses, then they will be treated, *as a matter of law*, as the responsible data controller. This has further fuelled the culture of caution for those who know that they are – or suspect that they might be – data controllers. The fact + law approach generates further anxiety and confusion within the research and governance communities. The lack of legal certainty (or mandate) hinders confidence within the system, preventing key actors from taking advantage of the flexibilities available to them.

All of this points towards a number of conclusions and focal points for further action. First, reform of the law is not a necessary first step to bring about reform of practice.^46^ The law already allows a lot to happen in terms of data linkage and sharing. Second, attention should be drawn to the spaces in-between legal provisions – where judgement calls must be made about data linkage. What is it that leads to a cautious approach for those operating within these spaces? Is it only the threat of future legal sanction or is it rather a lack of guidance and clarity on the purposes and values of data linkage itself? Third, wither privacy in all of this? That is, what mechanisms, if any, exist for decision-makers to weigh up considerations such as privacy risks and promotion of public interest through robust data linkage? Finally, who is being asked to take these sensitive decisions when the law might broadly permit discretion but offers little concrete guidance on how to proceed and justify each outcome? We suggest that these issues are the proper focus of regulatory attention and should be the subject of any models designed to deliver good governance. Crucial to success, however, is the further need to ensure that any models are responsive both to the needs of those whose data are being used (citizens) and those who are being governed (researchers themselves). This is something that, as the next section demonstrates, has not happened to date.

### Failure to incorporate iterative intelligent design

It is evident from our discussion that the current landscape has been constructed in fragmented and disjointed fashion, with little attention paid to the coalface of data linkage decision-making. This is particularly disappointing given the multitude of recommendations that have been made from notable reports and consultations, some of which we discuss in more detail below. Most worryingly, current frameworks have grown up piecemeal,^47^ lacking sustained attempts to engage the diverse spectrum of stakeholders who are affected by, and must work within, the regulatory environment. In sum, there has been a net failure to incorporate iterative intelligent design. Successful incorporation of such design involves a dialogical relationship whereby proposals advanced are continually sounded out amongst key stakeholders and most notably, amongst those charged with implementing them in practice. This allows key weaknesses to be identified, solutions sought and proposals subsequently reworked. This requires an ongoing reflexive approach towards successful development of a framework and one that we have employed as our dominant method in the development of our principled, proportionate governance model (PPGM) below.^48^ This has been constructed largely outside legislative discussions. These, we believe, largely miss the point about what is required to deliver good governance in this area.

### The limits of law

The preceding discussion should not be taken to suggest that no efforts have been made to ameliorate the situation. Some of the most notable attempts have centred on clarifying or supplementing key legislation in the area. For example, the Article 29 Working Party – an independent advisory body on matters of European data protection – has released pertinent guidance for the eHealth community, attempting to shed light on the key issues. In particular, it has issued advice relating to (i) the role and relationship between data custodians and data processors,^49^ (ii) processing of data relating to e-health records^50^ and (iii) the definition and role of consent in lawful data processing.^51^ The European Data Protection Directive is under review^52^ and attracting considerable scrutiny, particularly in light of the recent Albrecht Report that advocates that research involving health data should only be conducted with data subject consent, which should be ‘freely given, specific, informed and explicit’.^53^ The process for legislative intervention in this area is notoriously precarious.^54^ We avoid the temptation here to digress into detailed discussion of proposed changes, which, although of relevance to this topic, remain tentative and very likely to change. But more fundamentally, and absent an extreme volte face from the current legislative regime, the argument in this article is that legal reform is *not* required. It is, in many senses, a distraction. Instead, we posit that the broad parameters for delivering good governance are already laid down in the legal architecture and that more law is not the answer. What is required, however, is a deeper understanding of how to operate *within* those parameters and in keeping with the established data protection principles in both a robust and effective manner to give effect to the twin purpose of the law to promote responsible sharing whilst adequately protecting privacy. We advocate a detailed means to work through the delicate balancing exercise that must be performed when individual privacy interests are juxtaposed with public interests in the health research context. Importantly, much of this framework is about offering assistance to data controllers and decision-makers who operate in the regulatory spaces in between the legal architecture. Thus, whilst the broad legal rules and principles^55^ offer a framework for decision-making, they do not give much guidance to decision-makers on *how* to weigh up the considerations^56^ and arrive at justifiable decisions about data processing. In this sense, the value of our model serves as a complement to the legal framework in identifying room for manoeuvre within it and by assisting relevant parties to occupy such spaces responsibly. Legislative reform is unlikely to remove the role for key concepts such as consent, anonymisation and public interest. Whilst it might place emphasis more strongly on one rather than another, this article offers a defensible middle path through an ever-changing landscape.

As the above suggests, we must therefore be conscious of the limits of law. Whilst legislative reforms can make a significant improvement in clarifying obligations, they also have the potential to breed more confusion; a fear already expressed around the new legislative proposals.^57^ The ICO has warned:

it would have been preferable for the Commission to have developed one comprehensive data protection instrument whether a Regulation or a Directive. Given the two different instruments proposed, it is important for there to be as much consistency as possible between these instruments.^58^

This also raises an important point about the role of harmonisation within the regulatory landscape, and we argue here that our model facilitates a coherent approach to governance, which is nevertheless sufficiently adaptable for the different specific circumstances implicated by different types of data linkage. Legislation is but one (not always entirely effective) means of improving practice.

Extensive reviews of the regulatory landscape have included audits of current practice and consultations extended across the research and data sharing communities, resulting in recommendations for modification of the current framework. The Data Sharing Review, for example, highlighted the default ‘consent or anonymise’ approach and called for clarification of the legal framework, openness and transparency and the removal of unnecessary legal barriers, whilst simultaneously maintaining robust privacy protections.^59^ The AMS stressed the need for proportionate governance as a reaction to the overburdensome demands of the governance landscape, as outlined above.^60^ Most recently, the Caldicott Review has added a new principle to the pre-existing six Caldicott principles: ‘[t]he duty to share information can be as important as the duty to protect patient confidentiality’. The additional principle acknowledges outright the importance of sharing information, a duty often forgotten or which is seen as antagonistic to preserving confidentiality.^61^

We fully endorse these calls for clarity and proportionality and go far beyond them in offering substantive argument and examples about what proportionate governance can look like in practice. This is to ensure that the call to arms is not so abstract so as to render its key message otiose. An added advantage is that our approach can improve upon the governance landscape *as it stands*. It offers an account of the relative weight that can be placed on various operational governance devices within the legal architecture – such as consent, anonymisation, authorisation and public interest – in ways that inform current and future legislative processes by revealing deeper understanding about the interaction *between* these devices and arguments about their respective merits and demerits. In the final analysis, the objective is to offer concrete mechanisms for steering a path that simultaneously protects and promotes both private and public interests.

## Deliberative delivery: developing a PPGM

This section outlines the methods employed in developing our governance model. It appraises the key governance considerations that emerged from our research and that subsequently informed the development of our PPGM. The model was developed within the context of the Scottish Health Informatics Programme (SHIP).^62^ This was a Scotland-wide endeavour bringing together academia and NHS Scotland to develop an infrastructure to facilitate better the uses of health data for research. The interdisciplinary and collaborative nature of the project set the tone for the methodology with an emphasis from the start on working closely with a diverse range of institutions and individuals, enabling an iterative, discursive and multidisciplinary approach to developing a governance framework. Early and sustained stakeholder engagement was invaluable to identifying the key and diverse ethical, legal, social and practical issues implicated in delivering a more streamlined system. Data controllers and health researchers offered particularly important insights into trade-offs and accommodation of interests to be made when navigating the uncertain regulatory environment. This research user engagement proved to be crucial in the delivery of an effective, robust and adaptive model, one that is reflective of the needs and sensitivities of key stakeholders. Furthermore, given that all potential stakeholders cannot be identified from the beginning, it became readily apparent that an efficient governance model must be versatile enough to accommodate different stakeholder needs which are both present at the time of construction *and* which might subsequently emerge. Obvious examples include cross-sectoral and international level data linkage. A degree of foresighting was therefore also required to imagine future scenarios that would test the limits of any system and also secure its adaptability.^63^

The research commenced with the identification of key obstacles – actual and perceived – impeding research using secondary datasets specifically within (but not unique to) the United Kingdom, and more specifically, the Scottish context. An extensive literature review surveyed primary and secondary legislation and case law, good practice guidelines issued by professional bodies and recommendations emerging from consultation reports.^64^ Secondary literature also contributed to developing an understanding of the status quo.^65^ We documented the functions of key actors within the current framework, particularly researchers, data custodians and oversight bodies such as the PAC in Scotland^66^ and regulatory bodies such as the Information Commissioner. A detailed overview of the complex legal landscape is offered elsewhere.^67^ The following brief account of the key ethical and legal issues that emerged from our scoping exercise (and consequently influenced the construction of our PPGM) is pertinent for present purposes.

### Navigating the path: key considerations for proportionate governance

#### Privacy

Paying due regard to individual privacy is rightly one of the most dominant considerations within the current information governance framework. However, it is not the only consideration, as reflected by the non-absolute protection given to privacy under common law, statute and human rights regimes.^68^ Furthermore, it is often *perceived* risk to privacy that provokes disproportionate procedural burdens.^69,70^ And yet, the law, for the most part, does not adopt a risk-based approach to privacy protection.^71^

Privacy is a notoriously protean concept. Despite its well-recognised objective value as demonstrated by its protected status in a plethora of human rights and other legal instruments, it is – in fact – an inherently subjective notion for individuals. Opinion will vary considerably between individuals as to what *to them* constitutes privacy and its infringement. Often this will be highly context specific. In law, identifiability – that is, the likelihood of being able to identify an individual from their data – is often used as a key benchmark for triggering legal protection of privacy within the data sharing sphere, notably data protection. This, however, rarely involves an assessment of the nature or degree of the privacy risks or affront involved – which, again, will be dependent on context.^72^ Furthermore, identifiability is itself particularly problematic. Advanced technical procedures can ‘pseudonymise’ and ‘anonymise’ data, thus rendering re-identification of an individual highly unlikely, but it is impossible to guarantee 100% anonymity.^73,74^ The recent Caldicott Review acknowledges the problematic ‘grey area’ of data where re-identification of individuals is possible, particularly when combined with other data.^75^ The ICO stresses that 100% anonymity is not a requirement of the DPA and in its new Code of Practice, it is encouraging practitioners to view anonymisation as rendering the risk of re-identification remote rather than mitigating it completely.^76^ This reinforces the point that the risk assessment in determining adequate privacy protection is crucial.

Matters are complicated further by a tendency to conflate privacy with other concerns, most notably: autonomy, security, control^77^ and inaccessibility. With regard to privacy and autonomy, the latter tends to be determined by choice. It is not self-evident, however, that the same is true of privacy: simply because an individual has made a choice around whether she or he would like their information to be used (or not), this does not guarantee that their privacy concerns will be met. Privacy and security should also be differentiated: ‘security is necessary but not efficient for addressing privacy’.^78^ Privacy correlates with the use of data in that it implicates considerations of (mis)appropriate uses and typically relates to policies and procedures around data sharing. Security, however, relates to the protection that is afforded to data and relates more to operational and technical considerations.^79^

The essential nature of privacy interests also raises important considerations. For example, consider privacy and its relationship with the notion of control over information relating to each of us. Control can exist at different levels, for example, the individual level (via mechanisms including, but not limited to, consent). But individual level control does not guarantee privacy protection if data are to be shared in any form, because protection is also dependent upon external controls (such as privacy protection policies).^80^ Unless an individual chooses complete inaccessibility of their data (implausible in its own right), any consented use is dependent on external actors and policies around information handling. This in turn requires actors to avert to the privacy risks that will arise from the use itself – including ones that might not be known at the time any consent is given.

Whilst consent is not required under data protection legislation, under the common law duty of confidentiality, many regulatory and governance responses have proceeded on the assumption that some form of consent must be obtained prior to disclosure of personal information.^81^ This, however, has never been tested in the courts. Moreover, the need for informed consent in the context of secondary uses for data has been challenged with alternative solutions advanced. These include providing options for data subjects to opt out of studies where their data are used, providing one-off broad consent and including robust measures for ensuring privacy protection and safeguards to prevent indefensible infringements.^82^ Notwithstanding, there has been a conflation of consent concerns with privacy protection. A focus on consent has the double disadvantage that it distracts from the fact that privacy is not an absolute right, and it can be justifiably encroached upon in the public interest.^83,84^ Thus, as noted by Taylor, ‘[w]hile maintaining confidence in a health system might be an important public health objective … ’ – in that if patients do not believe their doctors will fulfil their obligations, they are less likely to step forward when infected – ‘… at the same time, health research is itself also an important part of protecting health generally’.^85,86^ To see privacy protection as an integral part of wider public health promotion is crucial. It allows meaningful comparison of the relative interests at stake in ways that are not possible if privacy is cast simply as a part of individuals’ autonomy interests. This is not to decry the importance of a role for consent, but it does require us to reorient our understanding of what is at stake. If public interest is a key and leading principle in guiding action in this area, then it requires a detailed account of its significance.

#### The public interest

Akin to privacy, the concept of the public interest is difficult to articulate across various realms of law, having attracted much attention from beyond the health sector.^87^ Whilst the notion remains ‘ill-defined’,^88^ public interest is perhaps more easily identifiable in the health context: the basic premise is that medical research using individual patient data can contribute to scientific knowledge that can be of benefit to the health of populations, individually and at large, now and in the future.^89^ We share a solidaristic, common concern in the protection and advancement of the public interest in health promotion,^90^ and this necessarily requires that in some circumstances individual and competing public interests must yield. Epidemiological studies or pharmacovigilance to identify drug risks are simply not possible without access to health-related data.^91^ If the premise is accepted, the challenge is to provide a defensible mechanism to conduct such trade-off exercises. Fundamentally, there is the need to show that there is indeed a public interest objective to be realised through a proposed data linkage. Given the primacy of privacy (and confidentiality), this places a significant onus on those claiming such a justification for data linkage and sharing. As a minimum, we contend that every linkage proposal would have to demonstrate that it is scientifically sound and that there are ethically robust reasons for the linkage. The strength of such a claim will be increased if it can be shown, for example, that only linkage with identifiable data will allow the public interest to be realised.^92^ But the spectre of consent is never far away: can public interest offer sufficient justification to proceed as an alternative to consent? More significantly, can public interest prevail even when consent is withheld?

#### Public interest and consent

We have asserted above and elsewhere that consent is often viewed as a panacea^93^ to all the risks brought up by data linkage. In the context of privacy protection, we have suggested that simply because an individual’s consent has been obtained, this does not guarantee that their privacy interests are being protected. As the dominance of consent in health-research regulation has been increasingly challenged in recent years, we have witnessed a morphing of the concept in an attempt to sustain its central role. Thus, we now have examples of consent being characterised as explicit,^94^ informed,^95^ specific,^96^ broad^97^ and generic.^98^ These last two examples are responses to the fact that it is often impossible^99^ or impracticable to provide individuals with information in all situations about what might happen to their information, especially when uses might be in the future and as yet undetermined,^100^ or when data that are to be used were obtained historically and for long-exhausted purposes.^101^ In the data protection arena, the European Article 29 Working Party has appreciated that consent does not always provide a strong basis for justifying the processing of personal data, particularly where consent is stretched to fit uses for which the consent was not initially provided.^102^ Why then does the appeal of consent endure?

An ambivalence about the relative roles of public interest and consent was borne out by our research that involved collaboration with colleagues in medical sociology who undertook engagement exercises on attitudes towards data sharing. These confirmed repeatedly the prima facie importance that people place in consent.^103^ Thus, albeit as a strict matter of law, public interest might prevail over consent, pragmatic and ethical considerations suggest that a more palatable practical approach would be to consider it a rebuttable presumption that consent ought to be obtained. This suggests that if the route is not to be taken, then strong justification and evidence for the public interest route is required. This re-enforces an important point about governance options: consent is not the only mechanism for justifying the use of patient data for research and public interest has a crucial role to play but their respective roles will depend on what is at stake. What is required, then, are mechanisms to assist researchers and data linkage decision-makers to reflect upon and ensure that the *appropriate* mechanisms are put in place for particular data uses in any given set of circumstances. It is far less about proscription of consent over public interest or vice versa as blanket positions and far more about constructing an appropriate governance response for particular linkage proposals. To do so, we must continue to consider the full range of the tools that is available in the governance armamentarium.

#### Anonymisation

A crude form of responsive governance has already prevailed in the perfunctory ‘consent or anonymise’ approach.^104^ Certainly, anonymisation can offer many advantages in the health research context. As Lowrance notes:

A way out of many problems should be de-identification, or anonymisation of data. If data are not identifiable the data are not ‘personal’ and, unless safeguards are compromised, the data-subjects stand only a very low risk of being harmed, which should be the principal point and should obviate the need for express consent. Much, perhaps most, health services research only uses anonymised data.^105^

Like privacy, however, this leaves us once again in the realm of non-absolutes. Anonymisation techniques render the likelihood of re-identification of data subjects highly unlikely^106^ but not impossible^107^ and the ‘grey area’ of data linkage can be particularly problematic.^108^ The ICO has acknowledged that potential for re-identification via data linkage is ‘essentially unpredictable because it can never be predicted with certainty what data is already available or what data may be released in the future’.^109^ Furthermore, anonymisation sets up a potential tension with public interest: whilst it takes us some of the way towards increased privacy protection, it can come at the cost of data quality; the richness or research potential value of data sets can significantly diminish once they have been anonymised.^110,111^ Equally, much valuable research can proceed without the need to use identifiable data.^112,113^ This leads to two important conclusions. First, like consent, anonymisation can serve a useful role as a default starting point from which departure is possible on good cause shown. Second, even if anonymisation is deployed in some form, risks remain. Thus, central to any good governance model must be appropriate risk assessment^114^ and the mechanisms and personnel to deploy this. This returns us, once again, to the option of authorisation.

#### Authorisation

As described above, authorisation involves an individual or group making a decision about whether data should be shared (and in what form) when it is neither possible nor practicable for the data subject to input to the process. Caldicott Guardians^115^ are the paradigm example of individuals who take on this role, whilst CAG in England and Wales and PAC in Scotland perform similar functions as collective bodies. Authorisation represents a move away from the binary ‘consent or anonymise’ model, but, importantly, it does not preclude a role for either consent or anonymisation in governance outcomes. That is, a conclusion from an authorisation deliberation might be that – in fact – consent should indeed be sought and/or that a certain form of anonymisation should be applied to the data. The important substantive and procedural value of authorisation lies in the opportunities afforded for deliberation, reflection, evaluation and risk assessment. Transparency and communication of such processes are also crucial in addressing possible issues of trust.

PAC in Scotland, for example, has an expectation that consent will be obtained where identifiable patient data are used. Whilst it recognises that this is not always possible, it holds that ‘in such circumstances, a clear explanation and justification should be given’.^116^ Amongst other things, explanations/justifications may include demonstrations of the scientific validity of a particular proposal, presentation of a strong case for why obtaining consent is not practical and evidence that privacy risks are minimised as far as possible and that adequate security measures are in place.^117^ Thus, authorisation provides a crucial piece of the governance puzzle by furnishing a means through which to determine the most appropriate mechanisms to be used and standards to be met for different linkage applications. This recognises that a one-size-fits-all approach is not appropriate. Notwithstanding, an important cross-cutting consideration for all data use and linkage applications is that of proportionality, that is, the robustness of justification of any authorisation must be relative to a sound assessment of the benefits and burdens involved.

#### Proportionality

Besides the key obstacles identified earlier, the most notable flaw tainting the governance landscape is a lack of proportionality. The landscape is ridden with disproportionate hurdles; many procedural mechanisms that researchers and data custodians must follow prior to data sharing and linkage often fail adequately to reflect the nature and degree of risks associated with such practices. As the Information Commissioner has said, the risks have been ‘both understated and overstated’.^118^ This incommensurability of regulation and risk – in either direction – becomes a matter of disproportionality when the regulatory burden greatly outweighs the relative risks. A failure to embody a sense of proportion in data linkage mechanisms naturally perpetuates a culture of caution and further impedes and delays progress with research.^119^ Thus, instilling and nurturing a more proportionate approach to governance that pays due regard to relative risks^120^ is crucial. This parallels one of the key recommendations of the influential Rawlins Report^121^ and the similar risk-based approaches being implemented by the Medicines and Healthcare Products Regulatory Agency with regard to clinical trials.^122^

Proportionality and its delivery by virtue of robust risk assessment are neither new concepts nor novel features of legal systems by any means. Proportionality plays an important role in European and human rights law, for example. Within the European Convention on Human Rights (ECHR) paradigm, any interference with many rights – notably private and family life under Article 8(1) – must be necessary and proportionate to meet a pressing social need under Article 8(2). Within European Law, the principle of proportionality is interpreted similar to a rationality test whereby the suitability, necessity and proportionality in its strict sense are considered against an alleged infringing measure.^123^ In both contexts, proportionality serves to regulate the spaces in between hard laws. It operates when discretion must be exercised and when varying interpretations or legal measures might be justifiable but depend on extensive variables that cannot be legislated for on a case-by-case basis. Rather, an appeal to proportionality determinedly requires that analytical judgement be performed; requiring appropriate consideration of material variables against a background of core objectives to be achieved. Thus, when matters of private and family life are engaged, the nature, extent and consequences of interference are judged relative to the nature, strength and importance of the social need. Proportionality acts as the weighing measure. Similarly, when judgements must be made about the propriety of data linkage, we contend that the nature, degree and likelihood of privacy (and other) impacts must be weighed relative to the strength of the reasons for seeking linkage at all, notably in the public interest – as robustly laid out in any given application for data linkage.

We discuss the specifics of these variables presently. Proportionality, however, has a central role to play in acting as a temper in two key ways in the information governance context and is an integral feature of our governance model. First, it complements the balance that must be sought between privacy protection in the public interest and the public interest in scientific enquiry and discovery by requiring a deeper account of what is actually at stake, most particularly by asking about the relative strengths of the interests and likely threats thereto. Second, it suggests that multiple and differential governance responses are the most fitting regulatory responses because different combinations of variables – strengths of privacy interests/risks versus strengths of public interests and benefits – will require different degrees of protection, sharing, oversight and, ultimately, sanction. In turn, this will require differing deployment of governance mechanisms, sometimes favouring consent when, say, sensitive data or contentious research questions are in play; sometimes favouring anonymisation when the research objective can be realised without recourse to identifiable data. Authorisation allows an important reflective and corrective input, promoting well-informed and risk-based assessment of the entire range of considerations, as well as mechanisms to give an account of deliberations and to communicate reasons and justifications. A rather trite appeal to balance is nonetheless enhanced by an appreciation through this account of the relative role and importance that each governance device brings and how they can be deployed alone or in combination. Finally, a commitment to balance ensures that no one principle or concern reigns supreme, albeit that evidence suggests that: ‘[i]t is undeniable that consent remains the primary policy device in legitimating medical research’.^124^

### Going fishing: a template for proportionate governance

Having identified the key hurdles to overcome and the overarching ethical and legal principles demanding continuous consideration, we offer here a template of key elements of governance that serves as a blueprint for what optimal governance might look like in the context of data linkage for research. This template provided us with a lens through which to focus comparisons between the current approach and any under consideration. This template was also an output of iterative design: not only does it capture emerging questions and concerns from the literature, but additionally it reflects expectations arising from engagement with stakeholders in the field.^125^

The template offers three key functions:

1. It provides a flexible yet bespoke means of identifying key elements that reflect the concerns of stakeholders and key issues highlighted within the literature;
2. It provides a means for identifying the strengths and weaknesses of any current regulatory framework and
3. It facilitates comparisons between a current and proposed model of governance in order to check whether, and if so how, any proposed model will improve upon the status quo. Moreover, it provides a basis against which the fitness of a future model can also be tested.

Table 1 offers an overview of our template for achieving optimal governance.

**Table 1.** Template for optimal governance.

Question	Key consideration(s) involved
Who are the key stakeholders and are they satisfied? (Are the right people engaged at an early enough stage in the governance process?)	Identifying and engaging with the various stakeholders within a regulatory framework means that buy-in and cooperation is much more likely, despite apparently conflicting interests.
In what ways does any model under consideration reflect a proportionate approach to governance?	Proportionality should be a key feature of any governance system, legally, ethically and practically. It avoids excessive and overly cumbersome procedures whilst paying due regard to real risks and seeking appropriate measures where fundamental obligations must be met.
Do all parties involved understand the implications of a particular model?	A major criticism of the current landscape is its complexity and the confusion that it generates amongst researchers and data controllers. Ensuring that all actors fully understand their obligations and are confident in exercising them is paramount to an effective governance system.
What vetting and training methods will be implemented by any model?	It is important to ensure that appropriate methods for ensuring that only adequately qualified individuals gain access to, and/or have responsibility for, data. This implies a need for effective training and accreditation in any governance regime.
Is there accountability within the model and who is accountable at each stage?	This requires articulation of key roles and responsibilities within the framework and proportionate sanctions to be in place for non-fulfilment.
How is the model monitored/regulated?	This implies overview of key legislative provisions, guidelines and oversight practices.
How does the model fare when subject to a Privacy Impact Assessment (PIA)?	It is recommended by the Information Commissioner’s Office that organisations carry out PIAs to identify privacy risks to individuals’ personal information in order to identify failures/strengths of a governance system in handling risks appropriately. It can encourage proportionate rather than conservative approaches towards risk.
How does the model reflect public expectations and impact on public confidence?	Engaging with the public, particularly in an initiative that involves sensitive personal information is key. Taking account of public expectations in a governance model can engender public confidence, even when this does not mean that all views become part of the model.
How does the current and proposed model sit within the legal order?	Compatibility of governance model with legal requirements and, even further, whether or not the model impedes/facilitates/makes optimal use of the legal provisions.

The considerations included in the template are by no means exhaustive; indeed, questions should be tailored as appropriate to the specific governance setting under consideration. For example, when considering cross-sectoral data linkage, one additional question might be: ‘how does the model accommodate or impede data sharing with other sectors?’ A further consideration could involve data sharing on an international level, for example, ‘how does the model deal with international data linkage, including ensuring adequate ethical and legal standards are identified and met?’ Moreover, the template is determinedly generic in that it can be applied within and across a range of data sharing sectors. The purpose of developing such an instrument is to outline the pertinent issues that must be addressed in governance design and that will remain uncovered by a simple literature review. Applying our template proved particularly fruitful in (a) identifying the differences between theory and practice, that is, what is assumed to take place in light of regulatory requirements and what are the practical realities of realising these demands and (b) teasing out the nuances within different settings/organisations and how they approach the implementation of the governance demands.

Encouraging a range of stakeholders to apply the template to their work setting offers a holistic multidimensional picture of current practice and related difficulties. Additionally, it unveils the specific needs of the very actors required to navigate the framework on a daily basis, rendering a proposed framework more likely to succeed in delivering good governance. Asking such questions is effective for assessing current regimes and comparing them against future proposals. As such, our template is akin to performing a *Governance Impact Assessment*. It is a process that helps to identify risks, options and opportunities that include, but go far beyond, concerns about privacy and anything that could be revealed by a privacy impact assessment alone.^126^

Developing and applying our template to the current regulatory landscape enabled us to identify key weaknesses within the framework^127^ and to clarify issues that needed to be addressed by any model to be developed in the future. Here, we outline the key themes that emerged and subsequently informed the construction of our proportionate governance model.

### The catch: key findings

First, our preliminary scoping of the literature asserted a discontent with the landscape, well demonstrated above. Engaging with key stakeholders about their experiences in practice confirmed this and served to highlight the most problematic areas encountered in practice, for example, the extent of the hurdles that researchers encountered when gaining approval for data access and the real and urgent need for clear and effective training. We worked closely with the public engagement team who carried out investigations into the attitudes of key actors including researchers and data controllers.^128^ A key message to emerge of import in constructing our model was the importance of having clear, accessible articulations of the legal obligations for different actors at different stages of data use; despite the inclusion of procedures for ensuring staff received training around their data handling responsibilities, confusion remained about specific obligations and how these could be fulfilled. This uncertainty is doubtlessly reflected in the wider community and perhaps confirmed by the fact that data breaches persist in that community. There is a tendency for breaches to attract extensive press coverage; shaking public and professional confidence^129^ across a range of sectors, particularly, where breaches occur within a trusted institution^130^ such as the NHS.^131^ Indeed, whilst recommendations have been made for Scotland^132^ and UK-wide^133^ public education campaigns to raise awareness amongst the public about how their data are used (including data about individuals who are relatively well),^134^ levels of understanding about data linkage and use are relatively low.^135^

Public engagement work within SHIP also uncovered the importance of trust in the safe and appropriate use of data.^136^ Thus, in our construction of a governance template, developing a model that reflected stakeholder and public concerns and attitudes was paramount. In particular, we appreciated that at the time of developing our model, a future move would be to develop initiatives that would facilitate cross-sectoral data linkage, that is, linking data across different sectors, for example, police, welfare, education and so on. Thus, any governance model must in many senses be both blind to sector-specific concerns and at the same time sensitive to them. This governance paradox was addressed by identifying the common concerns and pressure points for decision-makers across various sectors and designing governance tools of generic applications. Most notably, as discussed below, this led to the development of a set of transferable principles and instances of best practice that are both relevant and adaptable across any number of fields. The overarching commonality is the articulation and setting of gold standard benchmarks for data linkage and sharing that at the same time guide decision-makers. This can operate to gain buy-in from data custodians in different sectors to be willing to share data outwith their own organisations/sectors and at the same time achieve a degree of approximation of considerations, standards and approaches irrespective of the sectors within or across which linkage or sharing takes place.

Risk also emerged as a key consideration. It transpired that despite ICO guidance suggesting organisations should carry out Privacy Impact Assessments, not all organisations took this on board; a lack of joined-up governance across different organisations and sectors was evident, suggesting a suboptimal detection of privacy risks involved in data linkage. Of note, key issues that gave rise to the most confusion were the vague nature of the DPA 1998 and the varying interpretations that had been adopted of the European Data Protection Directive by virtue of the margin of appreciation it grants to member states. Specifically, doubts endured over issues such as when/whether consent is necessary, and what type of consent is appropriate (implied, informed, broad etc). The interoperability of statute and common law further added to the confusion, notably whether the duty of confidence requires an individual’s consent even when the DPA does not.^137^ Further still, the potentially tense relationship between the Freedom of Information (Scotland) Act 2002 and the DPA 1998 remains at times challenging to negotiate given that the former serves to make (official) information more freely available and the latter to limit access to (personal) information. Additionally, we must consider the difficulties around anonymisation mentioned earlier. Clear guidance on anonymisation, acceptable procedures for carrying out the process and the circumstances in which it was (un)necessary was lacking from both the UK DPA and at a European level.^138^ A key lesson learned is that despite the associated issues, both consent and anonymisation are important and must be central features in a good governance model. This is both with respect to minimising risks and because publics expect them to have a central role.

The juxtaposition of these findings with the outcome of our literature reviews led to the conclusion that consent and anonymisation should remain the *starting point* to consider within a proportionate governance approach. By the same token, we could assure decision-makers that they can depart from these mechanisms and use other routes on good cause shown, including where it would be disproportionate to attempt to deploy consent or anonymisation. This approach should serve to reassure publics as being one that it suitably couched in caution with respect to their autonomy and privacy interests but which also seeks to promote the important public interests in play and to strike a defensible balance of interests overall.

Another key lesson learned was the real need for coherent researcher and data custodian training. In addition to the understandable lack of comprehension of the complex legal and ethical landscape, vetting procedures are not robustly or uniformly applied across the health sector. Indeed, we learned that many data sharing relationships were based on trust and previous experience of sharing; a clear need for training and vetting was identified. Transparent and intelligible procedures not only to establish who is accountable but for what, and when, are essential. This was not so much a question of accountability and sanction at the level of the regulator – the ICO has now been granted increased powers to impose monetary penalties of up to £500,000 on those in breach of obligations^139^ – but rather this related to inter-institutional or personal accountability in data sharing arrangements at different stages of a data life cycle.

In sum, the application of our template allowed four key themes to emerge: (1) the need for clarification around which standards and values should be observed and how this can be achieved; (2) the need for a proportionate, risk-based approach to governance and how this might be operationalised; (3) the need for clarification around the roles and responsibilities arising from data sharing and clear lines of accountability and (4) the general need for training and accreditation around data-handling issues.

## Delivering principled proportionate governance

Our model comprises the following key elements that correspond directly with the key needs we identified in our research. Here, we elucidate how these needs can be met through: (1) guiding principles and best practice; (2) safe, effective and proportionate governance mechanisms; (3) a clear articulation of the roles and responsibilities of data controllers and (4) researcher training. We focus on the guiding principles and safe, effective and proportionate governance elements because they enable us best to convey the key message of this article – the importance of delivering *principled proportionate governance*.

### Guiding principles and best practice

From the outset, good governance demands an accessible articulation of the different values and standards against which individual and organisational activity will be assessed.^140^ Principles, by their very nature, offer the ideal medium for relaying these standards^141^ due to their flexibility; they can be adapted and implemented in a manner which best suits the level of decision-making taking place. Principle-based regulation (PBR) has enjoyed much attention lately, most notably within the financial sector. Its benefits can be translated to the data linkage context very well.^142^ Appropriately constituted principles are specific enough to convey the intention behind them, yet broad enough to leave room for interpretation as each case demands^143^and as has been noted, ‘we need ethical principles to “permeate” down to all levels’ of decision-making.^144^ In recognition of these strengths of principles, we have developed a set of guiding principles and instances of best practice (GPBP). These principles were the result of an iterative process and developed by a Working Group comprising of a diverse range of actors involved in data sharing and research.^145^

A key criticism of principles is that they are often vague in nature, failing to provide adequate or specific content on how different values ‘ … should be factored into decision-making processes, such as whether data should be made available for sharing, whether institutional arrangements are sufficiently robust to accommodate data sharing and whether appropriate governance mechanisms are in place for such sharing’.^146^ Thus, alongside the guiding principles, a set of instances of best practice were developed, offering more concrete examples of implementation of the principles.

Our approach stresses the importance of viewing principles not as quasi-rules, but as starting points for deliberation^147^ to exercise action and judgement within the existing legal regime. It openly acknowledges that principles might conflict and that discretion must be exercised in order to determine which set of principles should hold sway in the particular circumstance. The value of this approach is that it invites and requires reflection and justification. The principles that were identified were developed through engaging stakeholders on the issues and distilling these down to key principles that provided a common language for deliberation on whether and how sharing and linkage should occur. Self-evidently, the two principal principles at stake are: (1) promotion of the public interest and (2) protection of the privacy and other interests of citizens. A PBR approach would suggest that decision-makers strive to align as many principles as possible, for example, by promoting anonymised data to deliver robust research in the public interest. Where, however, this cannot happen, then other principles might come into play. In the example above, where anonymisation cannot happen or would unduly compromise the study, then it is a rebuttable presumption that patient consent should be sought.^148^ Where consent is neither possible nor practical, the principles call for authorisation from an appropriate body/research ethics committee.^149^

Developing a set of agreed principles is not necessarily a clear-cut or smooth process. It implicates all stakeholders within the regulatory landscape and as mentioned, must achieve a balance between versatility and specificity, whilst simultaneously remaining true to its goal of nurturing respect for the various considerations evoked by a particular framework. However, whilst the articulation and establishment of a set of principles is, in our view, an integral foundation for a good governance framework, principles are by no means intended to replace the role or content of legislation. Rather, they stress the values and norms to be considered *in addition to* the legislative demands upon different actors. Whilst the law provides flexibilities and space in between rules to exercise discretion, the principles provide a common framework^150^ for discussing and deciding what should be done,^151^ formed around the key considerations at stake. By the same token, guiding principles within a framework should not be regarded as optional or unimportant: they are the manifestation of key ethical norms and must be given due regard. This is so whether or not they engage legal sanctions for non-observation. Thus, achieving buy-in and endorsement from key actors is integral to the successful adoption of and respect for principles. Their generic nature can be a strength.

The Scottish Government adopted an iteration of our Guiding Principles and Best Practice for its Scotland-wide Data Linkage Framework for Statistical and Research Purposes.^152^ Such an endorsement and the proliferation that it guarantees across the Scottish research community demonstrate the importance placed on having an accessible and flexible expression of central values and standards for decision-making in the research context. The UK ICO has taken a similar approach to offering best practice guidance in its new code of practice for anonymisation.^153^ Similarly, to our GPBP, the ICO has made the purpose of the guidance explicit, in stressing that it is not designed to replace legislation (in this instance, the UK DPA), but rather to ‘plug that gap’^154^ between the minimal legal requirements set out by the legislation and the practical measures to take to facilitate compliance.

### Safe, effective and proportionate governance

Proportionality is a concept, which ensures that any measures taken (whether in terms of sanctions for breaches/non-observation of key standards, or anticipatory measures in place to assess risks within an organisation or across a regulatory landscape) correspond to the gravity of any breaches, actual or anticipated.^155^ But, it is not first and foremost about sanction. It is about matching the right governance pathway with the right risk assessment – long before there is a need to consider sanctions. This raises once again the central role that risk assessment plays in facilitating proportionate governance and the importance of a holistic approach to risk and which encompasses a range of risks that might include risk to privacy and reputation, or of distress to individuals through re-identification. SHIP has adopted such a holistic approach.

Our risk-based approach demands that certain benchmarks must be met before a holistic risk assessment is made. These benchmarks include seeking assurance on the following: safe data, safe people and safe environment. ‘Safe data’ involves data ‘adequately protected in a manner corresponding with its sensitivities, but this should not be to the extent that it renders data inaccessible or extremely difficult to access for important research purposes.’^156^ A host of considerations are engaged when assessing whether data are safe, including: whether consent is needed; whether a data reuse has been justified (particular where anonymisation is not practicable or desirable); the level of anonymisation, how disclosive the linked data may be, that is, how likely is it that individuals might be identified if the data are put in the public domain.

‘Safe people’ corresponds to the need for effective training of individuals and a clear articulation of the roles and responsibilities of different individuals throughout the course of the data life cycle. SHIP operates a researcher accreditation system. A ‘safe environment’ involves incorporation of sufficient security measures in order to ensure that data are safeguarded. For example, one must consider who has access to the data and in what circumstances the data may travel, if at all.

The paradigm example of this tripartite benchmark approach coming together is in one of the SHIP safe havens. This approach has been recommended by the Data Sharing Review, the AMS Report and most recently, the Caldicott Review. Much access is facilitated through these havens, which embody the three elements of safe data, people and environment and typify a form of principled, proportionate governance. This approach does not, however, work for all research, and so a more extended model also operates. Moreover, if any application fails on any of these three benchmark criteria, a full consideration by an authorising body is required.

In addition and directly related to the key benchmarks, we constructed a system that categorises different types of data access applications according to different categories of risk. In turn, these stratified risk categories correspond directly to increasingly stringent terms and conditions that must be met in order to achieve authorisation for a linkage to go ahead. The SHIP online toolkit (discussed below) helps researchers to anticipate the category in which their access application is likely to fit; this means, in turn, that the researchers can include the relevant details of relative risks associated with their study in anticipation of the terms and conditions to which an approval might be subject.

Categorisation is a manifestation of proportionality. For example, in situations where the privacy risks are minimal or negligible, and the likelihood of subsequent disclosure very small, no further review will be needed. Where risks are marginally greater, a fast-track process can be deployed that does not oblige a researcher to travel all the way to a safe haven to carry out the linkage. The highest risk applications must always be scrutinised by a suitably appointed authorising body. A Research Coordinator is responsible for advising from an early stage under which category an application should be made and a system of precedents will be built up over time. This further streamlines the processes and reduces undue burden in preparation of applications. Importantly, and reflecting the legal position, the data controller always retains the right to disagree with a categorisation and/or to refuse linkage or sharing in the final analysis.

**Figure 1.**
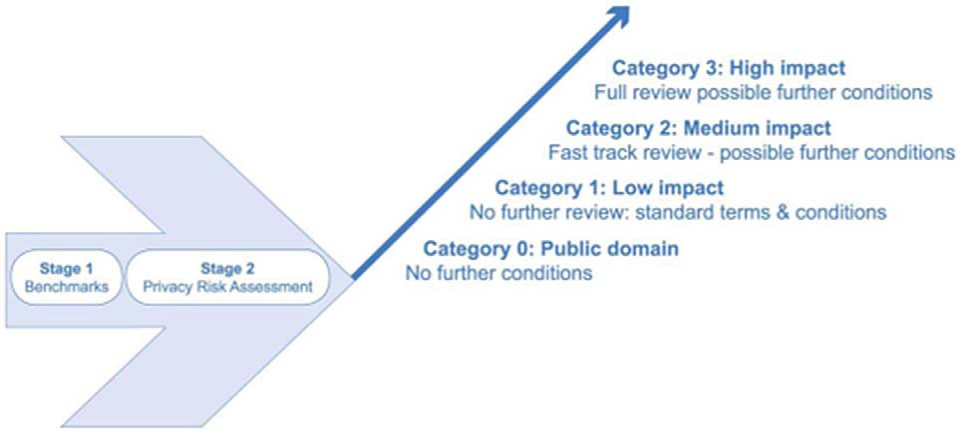
Categorisation of applications.

In sum, SHIP has taken a four-levelled approach to categorisation, inspired by the Understanding Society Project Data Access Strategy.^157^ This promotes further interoperability across sectors. The process is represented in Figure 1.

#### Category 0

This relates to data already in the public domain. Applicants are encouraged to make full use of such data, and these data are brought to their attention if research questions can be answered without the need to link personal or non-public data. This categorisation exercise might involve a prospective disclosure control exercise.

#### Category 1 **– **low impact

These applications are those where risks are thought to be minimal or negligible, and in particular, where outputs are non-disclosive and non-sensitive. Examples include those where no concerns are raised at stages one or two; the application is for a linkage which is non-disclosive and non-sensitive, a safe haven system^158^ will be used, and/or applications are for a non-contentious extension of a previously approved linkage.

#### Category 2 – medium impact

Category 2 applications are those where issues might be flagged for possible further consideration. These could be sent to the relevant advisory committee (in the case of SHIP, this would be PAC for Scotland) in an expedited form. Examples include applications with moderate risks or concerns arising from the privacy impact assessment at stage two; with repeat requests from multiple sector/international/researchers who are able to demonstrate a trusted track record with respect to SHIP and where the application is for a non-sensitive and non-disclosive linkage but safe haven system will not be used.

#### Category 3 – high impact

These applications would be subjected to full PAC approval mechanisms. Examples include applications that fail to satisfy any one of the criteria for assessment at stage one (e.g. questions over the public interest in the research, safe data, safe people or safe environments, or wider risks such as reputation of the data controller); raise concerns arising from the privacy impact assessment at stage two (e.g. very sensitive data; serious risks of disclosiveness) and/or are multiple sector or international linkages being requested for the first time.

In all cases, appropriate terms and conditions for sharing and linkage reflect the nature of the governance pathway followed by any given application and can be associated with different categories of applications. For example, category 3 might attract additional conditions about security or guarantees of no further linkages. Category 1 should be treated as standard linkages subject to everyday duties of confidentiality and institutional standards.

**Figure 2.**
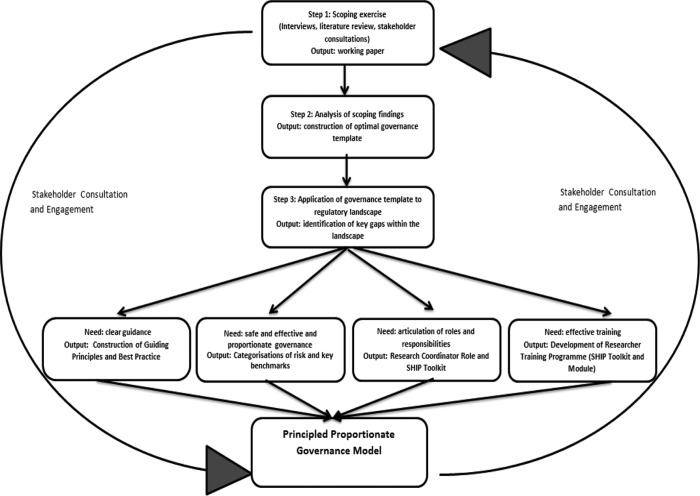
Methods for developing the principled proportionate governance model.

The categorisation approach is designed not only to offer a more proportionate approach to risk allocation but to harmonise and speed-up the review process, rendering the applications and approvals process more efficient for researchers and data custodians alike.^159^ It is sufficiently generic to be of interest and value across a range of data linkage scenarios, both within the health sector and beyond, and also inside and outside Scotland. The remaining two elements of the SHIP model are an online training and accreditation module and guidance on roles and responsibilities of data controllers. Thus, in sum, Figure 2 summarises the approach that was employed in developing the PPGM.

### Limitations and challenges for the model

As with all models, the SHIP approach has its limitations. It is important to note two points here. First, SHIP does not attempt to address the prevailing culture of caution by providing more certainty, at least in the first instance. If anything, it embraces the reality that delicate and, at times, difficult judgement calls about data linkage must be made. It might not, therefore, reassure some actors who seek more immediate clarity and security. We suggest, however, that such a search for security is illusory. What is needed, in particular, is a shift from defensive consent–reliant attitudes towards more confident data sharing and access, adopting a range of alternative mechanisms such as authorisation. It is hoped that the key elements of our approach, especially the training programme and the guiding principles, can serve to reassure researchers and data custodians when navigating the governance landscape.

Second, in order to operate effectively and timeously, there is a need for adequate resource and training for decision-makers themselves, including data controllers. It will only be through uptake and use of the system that streamlining benefits will be realised. In particular, the role of the Research Coordinator is crucial. This will often require additional resource, or redeployment, within organisations. If this resource is not put in place, delays will occur and time frames will not be respected. The aspiration of proportionality can only partly be delivered through iterative design. Its execution also requires committed personnel.

## Conclusion

The SHIP principled proportionate governance model has identified and seeks to address the pressing needs of research uses of health data, for which the current regulatory framework is both untenable and undesirable. The model challenges the traditional obstacles within the landscape, encouraging a shift away from the culture of caution and a willingness to take advantage of the flexibilities within the current landscape. It offers a data linkage regime that reflects iterative, intelligent design, taking into account both research and public expectations. Key elements of the model include (1) guiding principles and best practice, (2) safe, effective and proportionate governance, (3) an articulation of the roles and responsibilities of data controllers and data processors and (4) the development of a researcher training programme, including appropriate vetting procedures prior to sharing valuable data.

The research and collaborations under the SHIP project serve as a case in point that a flexible, accessible solution can be developed and adopted, not only in the health research setting but across sectors and across jurisdictions. The model builds upon existing approaches to information governance and goes far beyond them, whilst retaining due regard for the ethical and legal norms at stake. It offers a practical solution to moving forward in realising the potential benefits of data uses for health research and which can be implemented currently, rather than awaiting (yet further) legislative reforms. It recognises the important place of consent by making it a rebuttable presumption of research governance, whilst offering a clearer role for complementary governance mechanism, such as authorisation. These promote risk-based approaches and principle-based reflection, judgement and communication of decision-making. It is a model that is anticipatory in design, adaptable to future (imminent) developments in data linkage, most notably for cross-sectoral and international settings. Proportionality plays a central role in enabling decision-makers to undertake appropriate evaluation of risks and benefits. It helps to ensure that researchers and data custodians are not practising conservative data sharing out of fear of sanctions. At the same time, it acknowledges that sanctions that are imposed should be relative to the risks involved. The model determinedly complements any existing or future legal framework by seeking to fill the spaces within it. Principles and instances of best practice offer a means of universalisable deployment of relevant norms and values, promoting high standards of research across the data sharing life cycle, across diverse settings, with concrete examples of how these principles can be implemented. Most notably, this principled proportionate approach offers a concrete means of balancing both the public interests in health research and protection of patient privacy.

## Footnotes

1 See, for example, Academy of Medical Sciences, *Personal Data for Public Good: Using Health Information in Medical Research* (2011); Academy of Medical Sciences, *A New Pathway for the Regulation and Governance of Health Research *(2012); R. Thomas and T. Walport, *Data Sharing Review Report *(2008); W. Lowrance, *Learning from Experience: Privacy and the Secondary Use of Data in Health Research *(London, UK: Nuffield Trust, 2002); B. Miriovsky et al, ‘Importance of Health Information Technology, Electronic Health Records, and Continuously Aggregating Data to Comparative Effectiveness Research and Learning Health Care’, *Journal* *of* *Clinical Oncology *15 (2012), pp. 4243–4248; I. Kohane, ‘Using Electronic Health Records to Drive Discovery in Disease Genomics’, *Nature Reviews Genetics* 12 (2011), pp. 417–428; C. Safran et al, ‘Toward a National Framework for the Secondary Use of Health Data: An American Medical Informatics Association White Paper’, *Journal* *of the American Medical Informatics Association *14 (2007), pp. 1–9.

2 Such initiatives include: SHIP (Scottish Health Informatics Programme). Available at: http://www.scot-ship.ac.uk (accessed 2 December 2012); EHR4CR (Electronic Health Records for Clinical Research). Available at: http://www.ehr4cr.eu (accessed 2 December 2012); The Secure Anonymised Information Linkage (SAIL) Databank. Available at: http://www.ehi2.swansea.ac.uk/en/sail-databank.htm (accessed 2 December 2012).

3 Data Linkage WA. Available at: http://www.datalinkage-wa.org (accessed 14 September 2012).

4 Manitoba Centre for Health Policy. Available at: http://umanitoba.ca/faculties/medicine/units/community_health_sciences/departmental_units/mchp (accessed 14 September 2012).

5 Medical Research Council, E-Health Informatics Research Centres Call. Available at: http://www.mrc.ac.uk/Fundingopportunities/Calls/E-healthCentresCall/index.htm (accessed 29 August 2012).

6 As we have argued elsewhere, G. Laurie and N. Sethi, ‘Towards Principles-Based Approaches to Governance of Health-related Research using Personal Data’, *European Journal of Risk Regulation* 1(2013), pp. 43–57.

7 See Scottish Health Informatics Programme, Electronic Health Records for Clinical Research and The Secure Anonymised Information Linkage (SAIL) Databank in addition to: A Scotland-wide Data Linkage Framework for Statistics and Research: Consultation Analysis. Available at: http://www.scotland.gov.uk/Publications/2012/08/3287 (accessed 2 December 2012).

8 See in particular: B. Rumbold et al., *Understanding Information Governance: Access to Person-Level Data in Health Care* (London: Nuffield Trust, 2011); Academy of Medical Sciences, ‘*Personal Data*’ and ‘*A New Pathway*’; R. Thomas and T. Walport, ‘*Data Sharing Review *Report’.

9 J. Peto et al., ‘Data Protection, Informed Consent, and Research’, *British Medical Journal *328 (2004), pp. 1029–1030.

10 By virtue of the Health Research Authority (Establishment and Constitution) Order 2011, S.I. 2011/2323, the HRA has been established to protect and promote the interests of patients and the public in health research.

11 The Academy of Medical Sciences, ‘*A New Pathway*’, p. 6.

12 This is now known as the ‘ScottisH Informatics Programme’.

13 This work was supported by the Wellcome Trust through the Scottish Health Informatics Programme (SHIP) Grant (Ref WT086113). SHIP is collaboration between the Universities of Aberdeen, Dundee, Edinburgh, Glasgow and St Andrews and the Information Services Division of NHS Scotland. We would like to thank Wellcome for their support.

14 See notes 3 and 4. Particular studies from the Scottish context include L. Govan et al., ‘The Effect of Deprivation and HbA(1c) on Admission to Hospital for Diabetic Ketoacidosis in Type 1 Diabetes’, *Diabetologia* 55 (2012), pp. 2356–2360; J. Walker et al, ‘Effect of Socioeconomic Status on Mortality Among People With Type 2 Diabetes: A study from the Scottish Diabetes Research Network Epidemiology Group’,* Diabetes Care* 34 (2011), pp. 1127–1132.

15 Department of Health, *Information: To share or not to share? The Information Governance Review* (2013). See also Academy of Medical Sciences, *Personal Data*; R. Thomas and T. Walport, ‘*Data Sharing Review*’.

16 W. Lowrance, *Privacy, Confidentiality and Health Research* (Cambridge: Cambridge University Press, 2012) and ‘Learning from Experience: Privacy and the Secondary Use of Health Data in Research’, *Journal of Health Services Research* *and* *Policy* 8 (2003), pp. 2–7; D. Willison, ‘Privacy and the Secondary Use of Data for Health Research: Experience in Canada and Suggested Directions Forward’, *Journal of Health Services Research* *and Policy* 8 (2003), pp. 17–23; M. Law, ‘Reduce, Reuse, Recycle: Issues in the Secondary Use of Research Data’, *Spring IASSIST Quarterly* (2005), pp. 5–10; J. Brown and J. Semradek, ‘Secondary Data on Health-Related Subjects: Major Sources, Uses and Limitations’, *Public Health Nursing* 9 (1992), pp.162–171; P. Lelliot, ‘Secondary Uses of Patient Information’, *Advances in Psychiatric Treatment* 9 (2003), pp. 221–228.

17 See in particular: Academy of Medical Sciences, ‘*Personal Data*’ and ‘*A New Pathway*’; R. Thomas and T. Walport, ‘*Data Sharing Review*’; G. Laurie and N. Sethi, ‘*Current Practices*’ and ‘*Towards Good Governance*’.

18 House of Lords, Science and Technology Committee 2nd Report of Session 2008–09, ‘Genomic Medicine’ para 6.15. Available at: http://www.publications.parliament.uk/pa/ld200809/ldselect/ldsctech/107/107i.pdf (accessed 2 December 2012).

19 See in particular: Academy of Medical Sciences, ‘*Personal Data*’ and ‘*A New Pathway*’; R. Thomas and T. Walport, ‘*Data Sharing Review*’; G. Laurie and N. Sethi, ‘*Current Practices*’ and ‘*Towards Good Governance*’.

20 Directive 95/46/EC of the European Parliament and of the Council of 24 October 1995 on the protection of individuals with regard to the processing of personal data and on the free movement of such data Article 2(a) (hereafter referred to as the European Data Protection Directive).

21 C. Bennett and C. Raab, ‘The Adequacy of Privacy: The European Union Data Protection Directive and the North American Response’, *The Information Society: An International Journal* 13 (1997), pp. 245–264; Y. Poullet, ‘The Directive 95/46/EC: Ten years after’, *Computer Law and Security *22 (2006), pp. 206–217.

22 European Commission DG JFS, *Comparative study on different approaches to new privacy challenges in particular in the light of technological developments* (2010) p. 16.

23 House of Lords Science and Technology Committee, 2nd Report of Session 2008–09: *Genomic Medicine* (Stationary Office, London, 2009), para 6.15.

24 Recognised, for example, by the Information Commissioner: Information Commissioner’s Office, *Data Sharing Code of Practice* (Cheshire, ICO, 2011), p.4.

25 The UK Administrative Data Research Network, *Improving Access for Research and Policy: Report from the Administrative Data Taskforce* (2012), p. 16. Available at: http://www.esrc.ac.uk/_images/ADT-Improving-Access-for-Research-and-Policy_tcm8-24462.pdf (accessed 10 May 2013).

26 S. Harmon and K. Chen, ‘Medical Research Data-sharing: The “Public Good” and Vulnerable Groups’,* Medical Law Review* 20 (2012), pp. 516–539, at p. 522.

27 UK Information Commissioners Office, *Anonymisation: Managing Data Protection Risk Code of Practice* (Cheshire, UK: ICO, 2012) in Commissioner’s foreword.

28 Data Protection Act 1998, section 33.

29 ICO, *Code of Practice*, p. 46.

30 G. Laurie, ‘Evidence of Support for Biobanking Practices’, *British Medical Journal *337 (2008), pp. 186–187; G. Laurie and E. Postan, ‘Rhetoric or Reality: What is the Legal Status of the Consent Form in Health-Related Research?’ *Medical Law Review* (2012), pp. 1–44.

31 See, for example, General Medical Council, *Confidentiality* (2009) p.18 and ICO, *Code of Practice*, p.55

32 G. Laurie, *Genetic Privacy: A Challenge to Medico-Legal Norms* (New York, NY: Cambridge University Press, 2002), p. 335; N. Manson and O. O’Neill, *Rethinking Informed Consent in Bioethics* (Cambridge/New York: Cambridge University Press, 2007) p. 212; O. O’Neill, *Autonomy and Trust in Bioethics*, Gifford Lectures, University of Edinburgh (CUP, 2002).

33 R. Al-Shahi and C. Warlow, ‘Using Patient-Identifiable Data for Observational Research and Audit’ *British Medical Journal *321 (2000), pp. 1031–1032.

34 For a rejection of this approach and an argument for the need to see consent as process in the health research regulation context, see G. Laurie and E. Postan, ‘Rhetoric or Reality’.

35 Academy of Medical Sciences, ‘*Personal Data*’, p. 3.

36 ICO, *Anonymisation,* p. 13.

37 National Health Service National Services for Scotland (NHS NSS) Caldicott Guardians, see: http://www.nhsnss.org/pages/corporate/caldicott_guardians.php (accessed 2 December 2012).

38 G. Laurie and N. Sethi, ‘*Towards Good Governance*’, p. 16.

39 The Health Research Authority Confidentiality Advisory Group. Available at: http://www.hra.nhs.uk/hra-confidentiality-advisory-group (accessed 7 May 2013).

40 Previously the Ethics and Confidentiality Committee – see National Information Governance Board for Health and Social Care, Ethics and Confidentiality Committee. Available at: http://www.nigb.nhs.uk/ecc (accessed 3 December 2012).

41 CAG is also charged with advising the Secretary of State on non-research applications.

42 NHS National Services Scotland, Privacy Advisory Committee. Available at: http://www.nhsnss.org/pages/corporate/privacy_advisory_committee.php (accessed 3 December 2012).

43 Law, ‘Reduce Reuse Recycle’, p. 6

44 C. Kalman, ‘Increasing the Accessibility of Data’, *British Medical Journal *309 (1994), p. 740.

45 Directive 95/46/EC of the European Parliament and of the Council of 24 October 1995 on the Protection of Individuals with Regard to the Processing of Personal Data and on the Free Movement of such Data and UK Data Protection Act 1998

46 For example, see the recent ADT Report*, Improving Access*, which takes a ‘dual track approach’, acknowledging the difficulties that can be encountered when making legislative change and that ‘much can be achieved while the legislative timetable takes shape’ p.18.

47 MRC Ethics Series, *Personal Information in Medical Research* (London, UK: MRC, 2000).

48 We have argued for this elsewhere in the context of biobanks, see G. Laurie, ‘Reflexive Governance in Biobanking: On the Value of Policy Led Approaches and the Need to Recognise the Limits of Law’, *Human Genetics *130 (2011), pp. 347–356. More generally, see O. De Schutter and J. Lenoble (eds), *Reflexive Governance: Redefining Public Interest in a Pluralistic World* (Oxford, England/ Portland, Or: Hart Publishing, 2010).

49 Article 29 Data Protection Working Party, ‘Opinion 1/2010 on the concepts of “controller” and “processor”’, 00264/10/EN WP 169 (February 2010).

50 Article 29 Data Protection Working Party, ‘Working Document on the processing of personal data relating to health in electronic health records (EHR)’ 00323/07/EN WP 131 (February 2007).

51 Article 29 Data Protection Working Party, ‘Opinion 15/2011 on the definition of consent’ 01197/11/ENWP187 (July 2011).

52 See ‘Communication from the Commission to the European Parliament, the Council, the European Economic and Social Committee and the Committee of the Regions Safeguarding Privacy in a Connected World A European Data Protection Framework for the 21st Century’. Available at: http://eurlex.europa.eu/LexUriServ/LexUriServ.do?uri=CELEX:52012DC0009:en:NOT (accessed 19 September 2012).

53 European Parliament Committee on Civil Liberties, Justice and Home Affairs, ‘DRAFT REPORT on the proposal for a regulation of the European Parliament and of the Council on the protection of individual with regard to the processing of personal data and on the free movement of such data (General Data Protection Regulation) (COM(2012)0011 – C7-0025/2012 – 2012/0011(COD)), p.198. Moreover, it is argued that if consent is not possible then research should only be permitted if it served ‘an exceptionally high public interest’ and data must then be ‘anonymised or at least pseudonymised using the highest technical standards’.

54 Indeed, member states were given 3 years to transpose the Directive into national legislation, clear need for harmonised legislation was evident from the 70s and 80s – see Robinson et al., ‘Review of the European Data Protection Directive’ (2009) Prepared for the ICO. See: http://www.ico.gov.uk/upload/documents/library/data_protection/detailed_specialist_guides/review_of_eu_dp_directive.pdf (accessed 19 September 2012). Additionally, member states faced considerable challenges in implementing the Directive; most notably the European Commission claims that the UK Data Protection Act 1998 is a defective implementation of the Directive, claiming that it has failed to properly implement 11 of the 34 Articles.

55 T. Beauchamp and J. Childress, *Principles of Biomedical Ethics*, 5th ed. (New York/Oxford: Oxford University Press, 2001), at p.13.

56 M. Selgelid, ‘Universal Norms and Conflicting Values’, *Developing World Bioethics *5 (2005) pp. 267–273, at p. 269.

57 House of Commons Justice Committee*, The Committee’s opinion on the European Union Data Protection framework proposals*, Third Report (November 2012), HC paper HC 572.

58 ICO, *Information Commissioner’s Office: Initial analysis of the European Commission’s proposals for a revised data protection legislative framework* (February 2012), p.2. See: http://www.ico.org.uk (accessed 15 June 2013).

59 R. Thomas and T. Walport, ‘*Data Sharing Review*’.

60 Academy of Medical Sciences, ‘*New Pathways*’, p. 6.

61 Department of Health, *Information Governance Review*, at p. 62.

62 Now known as the ScottisH Informatics Programme; see further: http://www.scot-ship.ac.uk/ (accessed 15 June 2013).

63 On foresighting and law generally, see G. Laurie, S. Harmon and F. Arzuaga, ‘Foresighting Futures: Law, New Technologies and the Challenges of Regulating for Uncertainty’, *Law, Innovation and Technology *4 (2012), pp. 1–33.

64 A non-exhaustive list of sources we consulted included: Directive 95/46/EC of the European Parliament and of the Council of 24 October 1995 on the protection of individuals with regard to the processing of personal data and on the free movement of such data; Human Rights Act 1998; Data Protection Act 1998; Opinions from the Article 29 Data Protection Working Party; The Caldicott Principles; General Medical Council Guidance (particularly *Confidentiality*, 2009); NHS Act 2006; The Freedom of Information (Scotland) Act 2002 (FOISA), Academy of Medical Sciences, ‘*Personal Data*’ and ‘*New Pathways*’; Wellcome Trust reports and guidance from the Information Commissioner’s Office.

65 Again, a non-exhaustive list includes:, J. Kaye and S. Gibbons, ‘Mapping the Regulatory Space for Genetic Databases and Biobanks in England and Wales’, *Medical Law International* 9 (2008), pp. 111–130; W. Lowrance, ‘*Learning From Experience*’; J. Black, *Forms and Paradoxes of Principle Based Regulation*, LSE Law, Society and Economics Working Papers 13/2008 and *The ‘Principles’ Paradox*, Duke Law School Legal Studies Paper No. 205 (2008); S. Clark and A. Weale, *Information Governance in Health: An Analysis of the Social Values Involved in Data Linkage Studies* (London, UK: The Nuffield Trust, 2011) and T. Beauchamp and J. Childress, *Principles of Biomedical Ethics*, 6th ed. (New York: Oxford University Press, 2009).

66 This body performs a similar role to the Ethics and Confidentiality Committee in England and Wales, albeit that it does not operate under any statutory authority and is only concerned with linkage of data sets held by the Information Services Division of NHS National Services Scotland and the National Registers of Scotland (the bodies which set-up the Committee in 1990).

67 G. Laurie and N. Sethi, ‘*Current Practices*’ and ‘*Towards Good Governance*’.

68 For full discussion, see J.K. Mason and G. Laurie, *Law and Medical Ethics*, 9th ed. (Oxford: OUP, 2013), chapter 6.

69 For discussion on the effect of communication of risk by the media see: E. Singer and P. Endreny, *Reporting on Risk: How the Mass Media Portray Accidents, Diseases, Disasters, and Other Hazards* (New York, NY: Russell Sage Foundation, 1993); C. AbouZahr et al., ‘From Data to Policy: Good Practices and Cautionary Tales’, *The Lancet* 369 (2007), pp. 1039–1046.

70 For a broader discussion on newspaper reporting of medical records, see L. Brown, M. Parker and M. Dixon-Woods, ‘Whose Interest? British Newspaper Reporting of Use of Medical Records for Research’, *Journal of Health Services Research and* *Policy* 13 (2008), pp. 140–145.

71 This being said, the DPA 1998 does reflect a measure of risk escalation in that if sensitive personal data are involved then at least one condition of each of schedule 2 and 3 must be present. It is important to note that this does not necessitate consent nor does it suggest that autonomy trumps other considerations; rather it heightens safeguards for processing higher risk data.

72 An exception is found in the terms of the research exemption in section 33 of the Data Protection Act 1998, whereby ‘ … data must not be processed in such a way that substantial damage or substantial distress is, or is likely to be, caused to any data subject’.

73 P. Ohm, ‘Broken Promises of Privacy: Responding to the Surprising Failure of Anonymization’, *UCLA Law Review *57 (2010), pp. 1701–1777.

74 ICO, ‘*Anonymisation*’, p. 12.

75 Department of Health, ‘*Caldicott Review*’, p. 64.

76 ICO, ‘*Anonymisation*’, p. 13

77 See, for example, L. Bygrave, ‘The Place of Privacy in Data Protection Law’, *University of New South Wales Law Journal* 24 (2001), pp. 277–283.

78 L. Nakanishi, *The Difference Between Security and Privacy and Why We Must Better Communicate About Both*, Data Security and Privacy Group (2011); available at: http://datasecurity.edelman.com/the-difference-between-security-and-privacy-and-why-we-must-better-communicate-about-both/ (accessed 25 March 2013).

79 P. Thompson, ‘Privacy, Secrecy and Security’, *Ethics and Information Technology* 3 (2001), pp. 13–19.

80 For an interesting discussion on the need for separate notions of privacy and control see H. Tavani and J. Moor, ‘Privacy Protection, Control of Information, and Privacy Enhancing Technologies’, *Computers and Society *31 (2001), pp. 6–11.

81 Lord Falconer of Thoroton, ‘Privacy law and medical research’ [letter]. Times 2001 May 17:21, cited in Peto et al, ‘Data Protection’, p. 130. See also Information Commissioner’s Office, Data Sharing Code of Practice (Cheshire, ICO, 2011).

82 M. da Silva et al., ‘Informed Consent for Record Linkage: A Systematic Review’, *Journal of Medical Ethics *38 (2012), pp. 639–642.

83 Human Rights Act 1998 Article 8 Right to respect for private and family life. Article 8(1) Everyone has the right to respect for his private and family life, his home and his correspondence. Article 8(2) There shall be no interference by a public authority with the exercise of this right except such as is in accordance with the law and is necessary in a democratic society in the interests of national security, public safety or the economic well-being of the country, for the prevention of disorder or crime, for the protection of health or morals, or for the protection of the rights and freedoms of others.

84 ECHR Jurisprudence also demands that in order to prove interference is necessary and proportionate, the measure must demonstrate that (a) it addresses a ‘pressing social need’ (b) its operation is proportionate and (c) the reasons advanced for its existence are relevant and sufficient. See *Handyside v United Kingdom* [1976] 1 EHRR 737.

85 M. Taylor, ‘Health Research, Data Protection and the Public Interest in Notification’, *Medical Law Review* 19 (2011), pp. 267–303 at p. 277.

86 Department of Health, *Caldicott Review.*

87 For an informative account of the difficulties encountered with the public interest in the media, see House of Lords Select Committee on Communications, UK, *The future of investigative journalism, 3rd report of session 2010–2012*, HL paper 256 (31 January 2012), chapter 3. See generally, M. Feintuck, *The Public Interest in Regulation* (Oxford, UK: OUP, 2005), and more particularly and recently, see ‘Leveson Inquiry: Culture, Practice and Ethics of the Press’. Available at: http://www.levesoninquiry.org.uk/ (accessed 3 December 2012).

88 J.K. Mason and G. Laurie, *Law and Medical Ethics*, chapter 19.

89 J. Powell and I. Buchan, ‘Electronic Records Should Support Clinical Research’, *Journal of Medical Internet Research* 7 (2005).

90 B. Prainsack and A. Buyx, ‘Solidarity in Contemporary Bioethics: Towards a New Approach’, *Bioethics *26 (2012), pp. 343–350 and also ‘Solidarity: Reflections on an Emerging Concept in Bioethics’ (London, UK: Nuffield Council on Bioethics, 2011).

91 As Sheather notes, ‘confidentiality { … } serves a substantial public interest. A number of other significant public interests are in tension with this. A centralised health database offers enormous potential for research, which will itself feed into potential future health benefits. How should these interests be traded against each other?’. J. Sheather, ‘Confidentiality and Sharing Health Information’, *British Medical Journal *338 (2009), p. 1458.

92 The Caldicott Review acknowledges the legal basis of using identifiable data ‘exceptionally on public interest grounds’ however on the condition that linkage must only take place in an accredited safe haven. See *Caldicott Review*, p.65.

93 G. Laurie, ‘Evidence of Support’ and ‘*Genetic Privacy*’; G. Laurie and E. Postan, ‘Rhetoric or Reality’; N. Manson and O. O’Neill, ‘Rethinking Informed Consent’, and O. O’Neill, ‘Autonomy and Trust in Bioethics’.

94 M. Otlowski, ‘Tackling Legal Challenges Posed by Population Biobanks: Reconceptualising Consent Requirements’, *Medical Law Review *20 (2012), pp. 191–226.

95 A. MacLean, ‘From Sidaway to Pearce and Beyond: Is the Legal Regulation of Consent Any Better Following a Quarter of a Century of Judicial Scrutiny?’, *Medical Law Review *20 (2012) pp. 108–129.

96 M. Otlowski, ‘Tackling Legal Challenges’, p. 191.

 97 T. Caulfield and J. Kaye, ‘Broad Consent in Biobanking: Reflections on Seemingly Insurmountable Dilemmas’, *Medical Law International 10* (2009), pp. 85–100.

 98 T. Caulfield, ‘Biobanks and Blanket Consent: The Proper Place of the Public Good and Public Perception Rationales’, *Kings Law Journal* 1 (2007), pp. 209–226.

 99 For example, where the information related to incapacitated adults of children.

100 This might arise where patients cannot be traced or are deceased, see E. Regidor, ‘The Use of Personal Data from Medical Records and Biological Materials: Ethical Perspectives and the Basis for Legal Restrictions in Health Research’, *Social Science and Medicine* 54 (2004), pp. 1975–1894 at p.1976.

101 For example, where the participants group is so large that the study budget could not afford time or money to call each individual participant in order to obtain consent. See NHS NSS PAC (Guiding Principles and Policy for Decision-Making and Advice) and P. Furness and L. Nicholson, ‘Obtaining Explicit Consent for the Use of Archival Tissue Samples: Practical Issues’,* Journal of Medical Ethics* 20 (2004), pp. 561–564.

102 Article 29 Data Protection Working Party (2011) Opinion 15/2011 on the definition of consent; available at: http://ec.europa.eu/justice/policies/privacy/docs/wpdocs/2011/wp187_en.pdf (accessed 12 April 2013)

103 From the SHIP context see: M. Aitken, S. Cunningham-Burley and C. Pagliari, ‘Public Responses To The Scottish Health Informatics Programme: Preferences And Concerns Around The Use Of Personal Medical Records’, *Research Journal of Epidemiology and Community Health* 65 (2011): A27 and SHIP Public Engagement, ‘Your data and health research: SHIP Public Workshops’, ‘What makes researchers trustworthy’. Available at: http://www.scot-ship.ac.uk/publications (accessed 10 May 2013).

104 B. Rumbold et al., ‘*Access to Person-Level Data*’, p. 7.

105 W. Lowrance, ‘*Learning from Experience*’, S1, p. 5.

106 Ohm, ‘Broken Promises’, p. 1710.

107 See in particular Homer et al., ‘Resolving Individuals Contributing Trace Amounts of DNA to Highly Complex Mixtures Using High-Density SNP Genotyping Micro Arrays’, *PLoS Genetics* 4 (2008), pp. 1–11 and Ohm, ‘Broken Promises’, p. 1701.

108 Department of Health, *Caldicott Review*, p. 64.

109 ICO, *Anonymisation*, p. 18.

110 ‘100% anonymity is almost impossible to achieve without the data set being reduced to one data item, rendering it of little use for most research purposes’, Confidentiality and Security Advisory Group for Scotland (CSAGS), *Protecting Patient Confidentiality: A Consultation Paper, Seeking Consent* (2001). Available at: http://www.csags.scot.nhs.uk/ppc/ppc.pdf (accessed 3 December 2012).

111 ICO, ‘*Anonymisation*’, p. 13.

112 Ohm, ‘Broken Promises’, p.1701 and CSAGS, *Protecting Patient Confidentiality*.

113 The recent ICO Code on Anonymisation aims to clarify the conditions for acceptable levels of anonymisation and outlining when the process is necessary or desirable, whilst clearly acknowledging that such guidance is deficient both at the EU and UK level data protection legislation. Whilst it is too soon to say whether the Code is successful in clarifying to practitioners the issues around anonymisation, the extensive consultation and efforts that went in to the Code stand testament to how problematic anonymisation can be.

114 S. Shaw and G. Barrett, ‘Research Governance: Regulating Risk and Reducing Harm?’, *Journal of the Royal Society of Medicine* 99 (1994), pp. 14–19.

115 See Caldicott Guardians Forum. Available at: http://www.nhsnss.org/pages/corporate/caldicott_guardians.php (accessed 30 July 2013).

116 NHS National Services for Scotland, Privacy Advisory Committee for Scotland, ‘Guiding Principles’. Available at: http://www.nhsnss.org/pages/corporate/about_pac.php (accessed 30 July 2013).

117 In Scotland, in addition to PAC, the Community Health Index Advisory Group (CHIAG) also holds a key advisory role in relation to patient demographics and research uses. In each of these cases, the approach is similar: where consent or anonymisation are shown not to be viable options, the authorising body takes on a scrutiny role to consider the risks and benefits of linkage/use and to recommend an acceptable outcome. Where linkage is approved, then often additional terms and conditions can be imposed, for example, additional security measures or a reduction in access only to necessary data sets essential to answer the research questions.

118 ICO, ‘*Anonymisation*’, p. 3.

119 Academy of Medical Sciences, ‘*New Pathways*’, p. 6.

120 S. Shaw and G. Barrett, ‘Regulating Risk’, p. 18.

121 Academy of Medical Sciences, ‘*New Pathways*’, p. 52.

122 MRC/DH/MHRA, ‘Risk-adapted Approaches to the Management of Clinical Trials of Investigational Medicinal Products’ (2011). Available at: http://www.mhra.gov.uk/home/groups/l-ctu/documents/websiteresources/con111784.pdf (accessed 5 December 2012).

123 T. Harbo, ‘The Function of the Proportionality Principle in EU Law’, *European Law Journal *16(2) (2010), pp. 158–185.

124 Mason and Laurie, ‘*Law and Medical Ethics*’, chapter 19.

125 Key stakeholders within Scotland (and predominantly the SHIP community) included: researchers, data controllers, data custodians and data processors, and advisory bodies responsible for advising on data access applications for research.

126 ICO, Privacy Impact Assessment. Available at: http://www.ico.gov.uk/for_organisations/data_protection/topic_guides/privacy_impact_assessment.aspx (accessed 5 December 2012).

127 SHIP is a partnership between Information Services Division (ISD) of NHS Scotland and academic universities. We thus applied our template against the backdrop of the wider legislative framework within the UK and more specifically, procedures as they stood within ISD, custodian to the majority of NHS Scotland health information.

128 SHIP Public Engagement, ‘What makes research/researchers trustworthy?’.

129 B. Goold, ‘Technologies of Surveillance and the Erosion of Institutional Trust’, in K. Aas, ed. *Technologies of in Security: The Surveillance of Everyday Life *(Florida, FL, USA: Taylor & Francis, 2009) pp. 207–218 at p. 213.

130 UK Consumers Wake Up to Privacy. Available at: http://www.ico.gov.uk/upload/documents/library/data_protection/detailed_specialist_guides/icm_research_into_personal_information_feb08.pdf (accessed 3 December 2012).

131 K. Long, ‘Opinion Article: Public Confidence in NHS Integrity is Under Threat from Staff Breaches of Confidential Patient Information’ (2011). Available at: http://www.ehealthnews.eu/industry/2750-opinion-article-public-confidence-in-nhs-integrity-is-under-threat-from-staff-breaches-of-confidential-patient-information (accessed 3 December 2012).

132 Scottish Executive Health Department, ‘The use of personal health information in NHSScotland to support patient care’, *NHS HDL* 37 (2003), at p. 6.

133 See V. Armstrong et al., ‘Public Attitudes to Research Governance: A Qualitative Study in a Deliberative Context’, Wellcome Trust Report (London, UK: The Wellcome Trust, 2007).

134 Department of Health, *Information Governance Review*, at p. 62.

135 P. Singleton et al., *General Medical Council Public and Professional Attitudes to Privacy of Healthcare Data: A Survey of the Literature* (Cambridge Health Informatics Limited, 2007). See: http://www.gmc-uk.org/GMC_Privacy_Attitudes_Final_Report_with_Addendum.pdf_34090707.pdf (accessed 10 May 2013).

136 SHIP Public Engagement, *What makes research/researchers trustworthy?’.*

137 This issue gave rise to legislative change in England and Wales by virtue of section 251 NHS Act 2006 and the specific provision to allow the setting aside of the need for consent in specific defined circumstances under the authority of the Secretary of State (previously upon the advice of the Ethics and Confidentiality Committee, now the Confidentiality Advisory Group. Such legislative change has never occurred in Scotland. Instead the de facto approach of the Privacy Advisory Committee is similar to that of CAG, despite the lack of statutory powers, PAC advice carried considerable weight when advising ISD. ISDs of NHS Scotland are custodian of a vast range of NHS health data, and NRS is the National Register for Scotland.

138 Although we have mentioned that the ICO had now released its code of practice, it is hoped that this will provide much needed clarity, alongside the work of the UK Anonymisation Network. See: http://www.ukanon.net (accessed 11 May 2013).

139 As per powers enshrined under section 55 of the Data Protection Act 1998, the ICO can serve monetary penalties of up to £500,000 for breach of the Data Protection Act 1998 and serious breaches of the Privacy and Electronic Communications Regulations. At the time of writing, the ICO has served some 26 monetary penalties since January 2012, though note some of these have also been for breaches to Electronic Communications Regulations. See: http://ico.org.uk/enforcement/fines (accessed 9 May 2013).

140 Banff Executive Leadership Inc., ‘Improving Governance Performance: Rules-Based vs. Principles-based Performance’*, Leadership Acumen,* 16 (2004), pp. 1–5; Black, Forms and Paradoxes.

141 On the guiding principles of good governance, see Independent Commission on Good Governance, *The Good Governance Standard for Public Services *(2004), at p.4.

142 G. Laurie and N. Sethi, ‘Towards Principles-Based Approaches’.

143 T. Beauchamp and J. Childress, Principles of Biomedical Ethics, 6th ed. (New York: Oxford University Press, 2009); Seligman et al, ‘The Role of Values’; Financial Services Authority, ‘Principles Based Regulation’.

144 S. Arjoon, ‘Striking a Balance Between Rules and Principles-Based Approaches for Effective Governance: A Risk-Based Approach’, *Journal of Business Ethics* 68 (2006), pp. 53–82, at p. 55.

145 Further details and access to materials from this group can be available at: http://www.scot-ship.ac.uk/publications (accessed 10 May 2013).

146 G. Laurie and N. Sethi, ‘*Towards Good Governance*’, p.9.

147 See T Honderich (ed.), *The Oxford Companion to Philosophy* (Oxford, UK: Oxford University Press, 1995) at p. 719.

148 SHIP Guiding Principles: ‘Personal data must not be used without consent unless absolutely necessary’; ‘Where possible and practicable, consent should be obtained from each data subject prior to the use and sharing of personal data for research purposes’; and ‘Where personal data are used, the reasons and justification for its use are adequate and clearly explained.’ See SHIP Guiding Principles and Best Practice. Available at: http://www.scotship.ac.uk/sites/default/files/Reports/Guiding_Principles_and_Best_Practices_221010.pdf (accessed 10 May 2013).

149 SHIP Guiding Principle: where obtaining consent is not possible/practicable, then (a) anonymisation of data should occur as soon as is reasonably practicable and/or (b) authorisation from an appropriate oversight body/research ethics committee should be obtained.

150 N. Daniels, ‘Accountability for Reasonableness, *British Medical Journal* 321 (2000), p. 1300.

151 See, for example, J. Rawls, *A Theory of Justice* (Oxford: Clarendon Press, 1972).

152 Scottish Government, *Joined-Up Data for Better Decisions: Guiding Principles for Data Linkage* (2012). See: http://www.scotland.gov.uk/Resource/0040/00407739.pdf (accessed 30 July 2013).

153 See UK Information Commissioners Office, *Anonymisation: Managing Data Protection Risk Code of Practice* (Cheshire, UK: ICO, 2012).

154 Scottish Government, *Joined-up data*, at p. 10.

155 In enforcing its fines, the ICO does not differentiate between public and private organisations thus smaller research teams and organisations may lack the resources to pay such fines/would be radically affected by receiving them.

156 G. Laurie and N. Sethi, ‘*Towards Good Governance*’, p. 20.

157 Personal Communication – Economic and Social Research Council, Understanding Society Project, ‘Data Access Strategy’ Version 19.0. Note this is a draft and not currently available online. It is imperative that there be an approximation of approach within and across sectors to reduce regulatory burden and to help to ensure consistency of decision-making where this is possible.

158 For technical details on how SHIP operates, particularly the role of safe havens, consult the SHIP Blueprint. Available at: http://www.sco_ship.ac.uk/publications (accessed 10 May 2013).

159 For a more detailed account of the categorisation approach see G. Laurie and N. Sethi, ‘*Towards Good Governance*’, p. 35.

